# Rural Medical Education in New Zealand

**DOI:** 10.7759/cureus.826

**Published:** 2016-10-11

**Authors:** Heidi Mayer, Tia Renouf

**Affiliations:** 1 Department of GP and Rural Health, Dunedin School of Medicine, University of Otago; 2 Emergency Medicine, Memorial University of Newfoundland

**Keywords:** rural medicine, new zealand, education

## Abstract

Despite a large number of yearly medical graduates, rural New Zealand is faced with a scarcity of practicing physicians. Opportunities to learn and practice in rural settings start at the undergraduate level and extend to practicing physicians. There are a number of different programs available to facilitate rural medical education for all students and physicians. These programs will be discussed in this article.

## Editorial

Approximately 400 domestic medical students (excludes overseas students) graduate from the two New Zealand medical schools in Auckland (Auckland University) and Dunedin (Otago University) each year [1]. Although the number of graduates is increasing each year, there remains a shortage of rural practitioners [2]. Among the perceived disadvantages of rural practice are relative isolation coupled with onerous on-call duties, the lack of specialist training opportunities, and poor medical education.

Exposure to rural medicine occurs throughout the undergraduate curriculum. Opportunities to experience rural practice are available at the house surgeon level; postgraduate specialist training in rural and provincial hospital practice is a recognised training pathway. For doctors working in rural practice, as well as for those learning online, both regionally-provided education and specialist emergency medicine training (including trauma) are available, in addition to local and international conference opportunities. 

This article outlines these aspects of medical education for potential and current rural doctors.

### Undergraduate medical education

Both Otago and Auckland Medical Programmes have put in place initiatives to produce qualified doctors who are more likely to work in the rural sector. The Rural Origins Sub-Category admissions pathway (commenced in 2003) offers preferential entry to the students from rural backgrounds (determined by their residence and/or location of their primary/secondary school) who also meet academic admission standards and accounts for approximately 20% of each year’s entry [3].

The undergraduate medical course is six years long. Final exams are taken at the end of the fifth year, and the sixth year is spent consolidating clinical skills in general practice and hospital specialities. Exposure to rural medical practice begins in the second or third year while medical students are in the pre-clinical phase before they see patients. The students are either attached to a general practice in an observational capacity or spend a week in a rural town investigating health care facilities and speaking to a wide range of health providers, including general practitioners (GP), pharmacists, Maori health units, midwives, district nurses, and many others. 

More substantial time is spent in rural general practice during years four, five, and six. Students have placements for between two and six weeks, depending on which medical school they are attending. During these attachments, they spend most of their time supervised and mentored by a rural general practitioner. They are given their own room and carry out consultations before they are reviewed by their supervising general practitioner. Students experience the breadth and depth of rural practice, which includes palliative care, minor surgery, rest home visits, on call and emergencies which present at the surgery or in the community. During their time in practice, they also write up, present, and discuss several cases focussing particularly on community aspects of medical care.

Rural Medicine Immersion Programme

At both the universities, year five students may apply to study on the year-long regional-rural programme. This programme, which started in 2007, integrates primary, secondary, and tertiary medical care through real life experiential learning. During this year, students are guided and mentored by local and visiting tertiary hospital specialists, the Maori health faculty and providers, mental health teams, midwives, pharmacists, physiotherapists, rural general practitioners, rural hospital generalists, and rural nurses. The curriculum is delivered remotely and the use of information technology (IT) (audio and video conferencing equipment) is widespread, allowing frequent access to city-based specialist services and maintaining critical communication lines with fellow students. There is a strong emphasis on self-directed learning.

A recent Otago rural immersion programme student wrote:

“The biggest difference for the rural medical immersion programme (RMIP) is that it is really great for clinical training - more clinical time and more hands-on, but also much more self-directed, and less dedicated specialty teaching/exposure. We got far more clinical exposure than the city-based students, e.g. fifth-year students in Wellington have only three weeks’ hospital ward and community exposure to paediatric patients during the 10-week run, the remainder of the time spent in lectures or small group teaching [sic]. We had two half-day tutorials every week, and apart from that, we were in the hospital or GP clinic working with patients and with the consultants the whole time. 

What's more, there was only one of us at a time in the said clinical placement, so we were always lucky enough to be one-on-one with the consultant or GP. There were no house surgeons or registrars, which meant we got teaching from very experienced healthcare providers, and also had much more of a role to play, rather than just watching over someone’s shoulder and getting in the way!”

“There is always the worry with RMIP about getting enough specialty exposure as most of the centres are generalist, and about learning the right things for exams. However, I found that this was not the problem we thought it might be, as during the year you do end up seeing lots of the common presentations of each specialty and can follow their notes or even send them to the tertiary hospital if referred. There's also a lot of content that we are not directly taught of course, but this can be covered in textbooks such as the Canadian "Toronto Notes" that are very popular here and Oxford Clinical Handbooks, which is what I used.”

“To be honest, I chose RMIP because I wanted to spend the year mountain biking and skiing in Queenstown, New Zealand, but I would definitely choose to do it again, not only to spend time in a new place, but also for the wonderful one-on-one teaching opportunities, the much friendlier environment, the huge amount of clinical experience we get compared to the city-based students, and the fact that we had a role and were able to be part of the team and be useful, hardly ever just observing. I think it was really really beneficial to me, and I was lucky to do it.”

### Postgraduate medical education

House Surgeon General Practice

The postgraduate rural general practice training programme was set up in 2002 and offers a three-month run in a rural practice for Year 2+ postgraduate hospital doctors. The programme is run by the Royal New Zealand College of General Practitioners (RNZCGP), Wellington, New Zealand. It aims to expose junior doctors to rural general practice, rural medicine, and training outside the hospital setting, with the hope of influencing their future career plans towards work in rural general practice. During the attachment, they see their own patients, with a supervising GP on hand for advice and help when needed. The trainees are expected to see a diverse range of patients with a greater level of personal autonomy and responsibility for patients than in hospital settings. These experiences are designed to improve their understanding of primary care, and the importance of an effective GP-hospital interface. The attachment includes two hours protected teaching from an accredited teacher each week, which is funded by the RNZCGP, as well as protected time for study and learning.

General Practice Registrar

Doctors entering the General Practice Education Programme (GPEP) can choose to work in a rural practice, to undertake primarily practice-based learning, guided day-to-day by a GP teacher. In addition to all the usual clinical work, review of cases and consultations, one-on-one teaching and informal discussions, rural trainees take part in PRIME (Primary Response In Medical Emergencies) training (see below). 

PRIME Training

The PRIME programme is a jointly commissioned project funded by the Ministry of Health and Accident Compensation Corporation (ACC) – a government-funded insurance company covering all accidents. It is administered by St John’s, New Zealand’s ambulance service. It has been developed to provide both the coordinated response and appropriate management of emergencies in rural locations. The PRIME programme utilises the skills of specially trained GPs and/or rural nurses in areas to support the ambulance service where the response time for assistance would otherwise be significant or where additional medical skills would assist with the patient’s condition. The key objectives of PRIME are to support the ambulance service with a rapid response to seriously ill or injured patients and to provide higher level medical skills than may otherwise be available from the ambulance service in rural communities.

Rural GPs and nurses are initially required to undertake a five-day PRIME training course for trauma and medical emergencies. Then every two years they need to update their skills with a two-day refresher course. Both courses include theory and practical sessions including managing mock vehicle accidents and scenarios with multiple injuries.

Postgraduate Diploma in Rural and Provincial Hospital Practice

This is an advanced nationally recognised qualification for medical practitioners who staff rural and provincial hospitals. These doctors require both broad-based and specific skills, which may extend beyond that of rural general practice. The core papers include The Context of Rural Hospital Medicine, Communication, Obstetrics and Gynaecology, Surgical Specialties, Medical Specialties, Cardiorespiratory Medicine, and Trauma/Emergencies. A number of elective papers from a wide variety of topics, such as Maori Health, Travel Medicine, Ethics and Wilderness, and Expedition Medicine can be studied. This is a modular distance learning programme with online contact with tutors, regular assignments and essays as well as several two to three day seminars with lectures and workshops held at the university base. The programme usually takes two to five years to complete in part-time study. 

### Rural hospital medicine training

The Rural Hospital Doctor Training pathway provides vocational education for rural hospital doctors, which prepares them to work in the community’s rural hospitals. During this training, rural doctors acquire a core body of generalist knowledge as well as specific skills and attitudes that are needed to practice competently in a rural environment and a rural hospital. 

The training programme is divided into an academic programme, approved clinical attachments, and a final assessment. The academic programme is run either by the University of Otago (Diploma in Rural Hospital Medicine) or the University of Auckland (Diploma in Community Emergency Medicine). The trainees are also required to successfully complete Early Management of Severe Trauma (EMST), Advanced Trauma Life Support (ATLS) Level 7 and Advanced Paediatric Life Support (APLS) courses. The academic component is spread over three to four years.

The clinical training programme comprises a minimum of four years full-time equivalent in compulsory, recommended, and elective training attachments. The compulsory runs include one year in rural hospital medicine and three or six-month runs in general medicine, emergency medicine, paediatrics, anaesthetics/ICU, and rural general practice.

The Division of Rural Hospital Medicine New Zealand (part of the RNZCGP) accredits the clinical attachments, facilitates education, and provides processes to ensure clinical skills have been acquired. It also administers the final assessment for fellowship (vocational recognition).

Before entering into rural hospital medicine training, doctors have to have completed at least two full-time equivalent years of appropriate medical experience after graduating. This can include rural hospital and rural general practice runs. On entering the programme, the registrar is assigned an educational facilitator (a vocationally registered rural hospital doctor). The registrar and educational facilitator produce a professional report and training plan. The report identifies areas of prior learning and learning needs. The training plan will include intended clinical attachments, academic qualifications needed to meet the registrar’s identified learning needs, and requirements for fellowship.

There is considerable flexibility both at the entry and exit from rural hospital training and practice. Accreditors make it possible to train concurrently in, for example, rural hospital medicine and another generalist scope of practice such as general practice or emergency medicine.

### Does rural medical training result in more rural doctors?

A retrospective cohort study of 2008–2011 Otago Medical School graduates found that significantly more students who either had a rural background on entry into medical school or who spent a year training in the Rural Medical Immersion Programme, were training in the rural hospital medicine or rural general practice after graduation compared with other students [3]. In this study, 112 of 733 (15.3%) students had rural background/training. Multiple logistic regression identified both variables (rural background and rural training) as independently statistically significant (odds ratios (OR) (95% confidence interval (CI)); rural background OR2.1, 95% CI 1.2–3.6; rural training OR2.5, 95% CI 1.4–4.5; p = 0.002). In addition, almost twice as many students with rural background/training were working rurally (i.e. in non-major urban centres). However, because of the relatively recent adoption of the two rural initiatives, graduate data were only available for up to five years and differences were based on small numbers.

At the same time that this study was published, the RNZCGP undertook its 2014 workforce survey [4]. This provides a snapshot of the New Zealand Rural General Practice workforce (response rate 55.9%) with 17.1% of GPs identifying themselves as rural. Rural GPs were more likely to be male (57.5% rural vs 45.5% urban: p < 0.01), older (≥ 55 years) (38.2% rural vs 32.6% urban: p = 0.04) and international medical graduates (52.8% rural vs 38.7% urban: p < 0.01).

### Summary

Exposure to rural medical education starts in the undergraduate medical course with students spending time in rural general practice. Some students have the opportunity to study for one year on the Rural Medicine Immersion Programme as part of their undergraduate studies. Experience in rural medicine can be acquired as a junior doctor and at registrar level in general practice training. There is a postgraduate diploma in rural and provincial hospital practice and vocational training in rural hospital medicine for rural hospital doctors.

It appears that recent initiatives of preferential entry into medical school for students from rural backgrounds and undergraduate rural training are influencing postgraduate training choices and practice location. However, these programmes need to be sustained if the composition of the current rural medical generalist workforce is to change.
